# Prevalence of adult attention-deficit/ hyperactivity disorder symptoms among university students and their relation to sleeping duration, time management skills, and academic performance

**DOI:** 10.7717/peerj.21688

**Published:** 2026-10-05

**Authors:** Maged El-Setouhy, Suhaila A. Ali, Mohammad Zaino, Anwar Alahmar, Zakaria Y. Alsanosi, Sattam Jaddoh, Nadim I. Areshy, Abdulrahman H. Basheer, Ali A. Dagreri, Mohsen Y. Moafa, Rakan M. Hakami, Nasser Y. Idris, Ahmad H. Makeen, Ahmad Y. Alqassim

**Affiliations:** 1 Department of Community, Environmental and Occupational Medicine, Faculty of Medicine, Ain Shams University, Cairo, Egypt; 2 Department of Family and Community Medicine, Faculty of Medicine, Jazan University, Jazan, Saudi Arabia; 3 Physical Therapy Department, Faculty of Nursing and Health Science, Jazan University, Jazan, Saudi Arabia; 4 Jazan Health Cluster, Eradah Hospital And Mental Health, Jazan, Saudi Arabia

**Keywords:** GPA, Academic performance, Adult ADHD symptoms, Time management skills (TMS), Sleep duration

## Abstract

**Background:**

Attention-Deficit/Hyperactivity Disorder (ADHD) is a common neurodevelopmental condition that often persists from childhood into adulthood and is characterized by symptoms of inattention, hyperactivity, and impulsivity. In university students, these symptoms may negatively affect time management and academic performance. This study aimed to estimate the prevalence of adult ADHD symptoms among university students at Jazan University, Saudi Arabia, and to examine their associations with time management skills and academic performance.

**Methods:**

We conducted a cross-sectional study at Jazan University, Jazan, Saudi Arabia. We used a multistage sampling technique. We distributed 763 online questionnaires to the university students; the response rate was 87% (664 questionnaires). Of them, 612 (80.2%) questionnaires were complete and included in the analysis. We recruited male and female students from the Colleges of Medicine, Nursing, Business Administration, and Engineering. Data were collected using validated self-administered questionnaires assessing ADHD symptoms, time management skills, and academic performance.

**Results:**

Our study was conducted among Jazan University students, with a mean age of 21 years (SD = 1.6), and a predominance of male participants (64%). The prevalence of adult ADHD symptoms was 26% among Jazan University students. Significant associations were observed with female sex (odds ratio (OR) = 1.58), sleeping fewer than 4 hours per night (compared with ≥ 8 hours; OR = 2.46), and lower grade point average (GPA) (odds ratio (OR) = 2.10). Associations with the Academic Performance Scale (APS) and time management skills were inconsistent.

**Conclusions:**

Adult ADHD symptoms were more prevalent among Saudi university students than estimates reported in the global literature and were associated with female sex, insufficient sleep, and lower GPA. The inconsistent associations with academic performance measures and time management skills highlight the complexity of ADHD-related functional outcomes. These findings underscore the need for targeted interventions to support students’ mental well-being and academic success.

## Introduction

Attention-Deficit/Hyperactivity Disorder (ADHD) is one of the most common neurodevelopmental conditions typically diagnosed during childhood, though it frequently persists into adulthood (Baji et al., 2023; Drechsler et al., 2020; Hill & Schoener, 1996). This condition significantly impacts daily functioning, occupational performance, and interpersonal relationships (Bjerrum, Pedersen & Larsen, 2017; Fuermaier et al., 2021). While much literature focused on ADHD in pediatric populations, recognition and awareness of adult ADHD in adulthood have increased substantially over recent decades (Abdelnour, Jansen & Gold, 2022; Song et al., 2021).

The prevalence of adult ADHD symptoms varies considerably across studies, largely due to differences in diagnostic criteria, assessment methods, and population characteristics (Hill & Schoener, 1996; Song et al., 2021; Williams et al., 2023). Globally, estimates of prevalence of adult ADHD symptoms range from approximately 2.5% to 4.4% (Ayano et al., 2023; Song et al., 2021). Marked regional variation has been documented, with higher prevalence rates reported in North America (approximately 4.4–5%) and comparatively lower rates observed in Europe (2.5–3%) (Salari et al., 2023; Simon et al., 2009). In Arab countries, earlier studies reported ADHD prevalence estimates from 1.2% to 16% (Alhraiwil et al., 2015). More recent evidence suggests that prevalence of adult ADHD symptoms in the region remains heterogeneous and contested. For example, prevalence estimates in the United Arab Emirates range from 6% to 9%, whereas studies from other Arab countries report rates between approximately 4% and 6% (Al-Yateem et al., 2024; Aljadani et al., 2023).

In Saudi Arabia, reported prevalence estimates of childhood ADHD vary widely, ranging from 2.9% to 52.5% (Al-Saedi et al., 2023; Aljadani et al., 2023; AlZaben et al., 2018). In contrast, estimates of prevalence of adult ADHD symptoms remain limited and highly controversial. Recent studies have reported prevalence rates ranging from 11.9% to 46.6% among Saudi adults (Alghamdi et al., 2022; Alharbi et al., 2023; Alsafar, Alsaad & Albukhaytan, 2024). This substantial variability may be attributed to differences in study design, data collection methods, screening instruments, and sampled populations. In addition, the diagnostic complexity of adult ADHD and the subjectivity inherent in adult diagnostic criteria likely contribute to inconsistent prevalence estimates (Bogdanska-Chomczyk, Majewski & Kozlowska, 2025; Musullulu, 2025).

Historically, ADHD was considered to predominantly affect males; however, evidence indicates that this gender disparity narrows considerably in adulthood (Gilbert et al., 2023; Quinn & Madhoo, 2014; Skogli et al., 2013; Stibbe et al., 2020). Adult ADHD is also frequently associated with psychiatric comorbidities, including anxiety disorders, depression, and substance use disorders, which may mask ADHD symptoms and lead to underdiagnosis and underestimation of true prevalence (Katzman et al., 2017; Kooij et al., 2010). This ongoing controversy highlights the need for more methodologically rigorous and contextually grounded research.

Effective time management is a critical determinant of quality of life and academic success, particularly within higher education settings (Aeon, Faber & Panaccio, 2021). ADHD, characterized by deficits in attention regulation, impulse control, and executive functioning, is associated with impaired time management skills, which in turn contribute to poor academic performance and unhealthy lifestyle behaviors (Aeon, Faber & Panaccio, 2021; Espanol-Martin et al., 2023; Lauder, McDowall & Tenenbaum, 2022; Sobanski et al., 2013). Furthermore, academic stressors commonly encountered by university students may negatively affect their time management skills (Al-Shahrani et al., 2023; Barbayannis et al., 2022; Wahid et al., 2023).

Nevertheless, a recent narrative review reported substantial inconsistency in the literature regarding the relationship between adult ADHD and time management skills, highlighting mixed findings and considerable methodological variability across studies (Mette, 2023). Although executive dysfunction, particularly impairments in planning, organization, and sustained attention, has been proposed as a central mechanism underlying poor time management skills in ADHD, empirical evidence remains inconclusive (Durand, Arbone & Wharton, 2020; Grinblat & Rosenblum, 2025).

Several studies have shown that both adult ADHD and impaired time management skills are independently associated with poorer academic outcomes among university students, including lower academic achievement, increased academic stress, and reduced task completion efficiency (Arugaslan, 2024; Henning, Summerfeldt & Parker, 2022). These academic challenges are often attributed to difficulties in prioritization, meeting deadlines, and effective study planning.

Despite these findings, the interrelationships among adult ADHD, time management skills, and academic performance remain insufficiently understood. Additional qualitative and mixed-methods research may help clarify how adaptation and compensatory strategies influence academic functioning among university students with ADHD symptoms (Canela et al., 2017). Moreover, the predominance of research conducted in Western populations limits the generalizability of existing findings (Ben-Dor Cohen et al., 2024; Ginapp et al., 2023). Consequently, evidence addressing these associations within Middle Eastern contexts, particularly in Saudi Arabia, remains scarce.

Sleep duration was also examined in the present study, as sleep deprivation has been shown to exacerbate adult ADHD symptoms and impair cognitive processes such as concentration, organization, and task completion efficiency (Floros et al., 2021; Lim & Dinges, 2008; Saletin et al., 2019). Existing evidence suggests a complex and incompletely understood relationship between ADHD, sleep duration, and circadian rhythm disturbances (Gomes-Tiago et al., 2016; Luu & Fabiano, 2025). While these associations have been extensively studied in children, they remain underexplored in adult populations, particularly within the regional context.

Accordingly, this study aimed to examine the interrelationships among adult ADHD, time management skills, academic performance, and sleep duration among students enrolled at Jazan University during the 2023–2024 academic year.

## Hypothesis

As a null hypothesis, we posit that there are no significant relationships between ADHD symptoms, time management skills, sleep duration, and academic performance among university students.

## Materials and Methods

### Study design and setting

This cross-sectional study was conducted at Jazan University, a public higher education institution located in the southwestern region of Saudi Arabia, during the 2023–2024 academic year. The study targeted undergraduate students of both genders enrolled across the university’s various colleges. All participants were Saudi nationals, and no exclusion criteria were applied based on race or ethnicity.

### Participants and sampling

Participants were recruited from four colleges selected to represent the 16 colleges at Jazan University. The minimum required sample size was calculated using the Raosoft online sample size calculator (Raosoft, 2004), based on a total student population of approximately 60,000, a 95% confidence level, a margin of error of 5%, and an anticipated nonresponse rate of 40%. This yielded a minimum sample size of 367 students. To account for anticipated defaulter rate of 20% due to incomplete questionnaires, the target sample size was increased to 440 students. To improve precision and account for potential nonresponse, questionnaires were distributed to 763 students in the selected colleges.

A multistage random sampling technique was employed to ensure representation across academic disciplines. University colleges were first stratified into four sectors: medical (College of Medicine), paramedical (four colleges), scientific (four colleges), and literary (seven colleges). Using simple random sampling, one college was selected from each sector. The selected colleges were the College of Medicine, College of Nursing, College of Engineering, and the College of Business Administration. Subsequently, two classes (one male and one female, reflecting the gender-segregated academic structure) were randomly selected from each college. Questionnaires were distributed to approximately 180–200 students per college.

### Data collection

Data collection was conducted using an anonymous, self-administered, and assisted questionnaire consisting of 57 items organized into four sections. A team of trained investigators distributed the questionnaires along with informed consent forms to students from the targeted colleges. The distribution took place at the beginning of the students’ break time, during which the investigators provided a concise overview of the study’s purpose, methodology, potential benefits and risks, and emphasized the voluntary nature of participation. Our team instructed participants who were currently receiving treatment for ADHD or had previously received treatment for ADHD not to complete the questionnaire. Students who agreed to participate were instructed to provide their informed consent by selecting “I agree” before completing the questionnaire. Those who chose not to participate were asked to check “I disagree” and refrain from filling out the form. The investigators remained available in the classrooms to respond to any questions and collected the questionnaires upon completion.

A total of 763 questionnaires were distributed, of which 664 were returned (87%). After excluding incomplete questionnaires, 612 (80.2%) questionnaires were included in the final analysis (Fig. 1).

**Figure 1:**
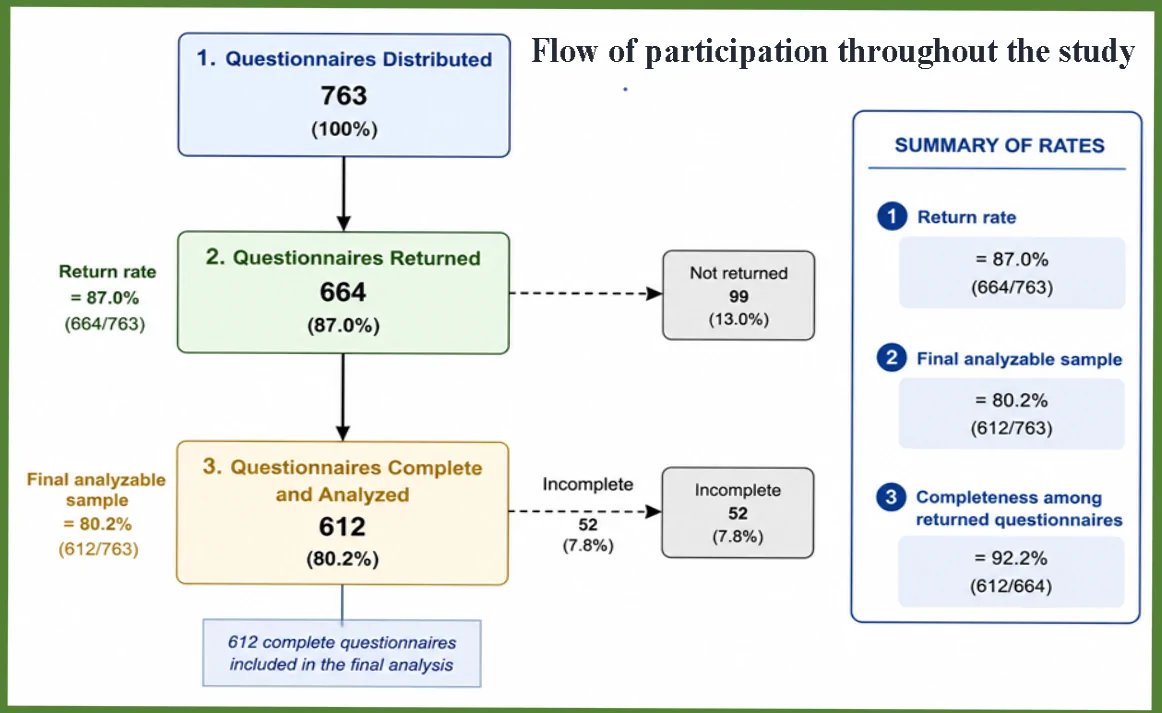
Flow of participants throughout the study.

### Study instruments and variables

#### Sociodemographic and academic variables

The first section collected data on age, gender, college affiliation, weight, height, sleep duration, monthly family income, father’s education level, mother’s education level, and grade point average (GPA).

#### Adult ADHD symptoms

Adult ADHD symptoms were assessed using the adult ADHD Self-Report Scale (ASRS-v1.1), a validated screening instrument developed in collaboration with the World Health Organization (WHO) and based on the Diagnostic and Statistical Manual of Mental Disorders, Fourth Edition (DSM-IV) criteria. The ASRS-v1.1 is a widely used screening tool designed to identify symptoms suggestive of adult ADHD; however, it is not intended for diagnostic purposes. The instrument consists of an 18-item symptom checklist. Of these 18 items, six constitute Part A of the ASRS-v1.1 Screener, while the remaining 12 items comprise Part B (Caci, Didier & Wynchank, 2024; Green et al., 2019).

The six items in Part A are rated on a 5-point Likert scale ranging from 0 (never) to 4 (very often) and have been identified as the most predictive of symptoms consistent with adult ADHD. Accordingly, the maximum possible score for Part A is 24. For screening purposes, the WHO recommends dichotomizing responses into negative (0) and positive (1) categories using item-specific thresholds. For the first three items, responses in the highest three categories are considered positive, whereas for items 4 and 5, responses in the highest two categories are considered positive. A total of four or more positive responses was considered highly indicative of adult ADHD symptoms. Part B consists of 12 items that assess the severity of inattentive and hyperactive/impulsive symptoms (Adler et al., 2019; Kessler et al., 2005; Stanton, Forbes & Zimmerman, 2018). In the present study, the ASRS-v1.1 demonstrated good internal consistency, with a Cronbach’s alpha coefficient of 0.84.

#### Time management skills

Time management skills were assessed using the validated Arabic version of the Time Management Questionnaire (TMQ) developed by California Baptist University (CBU, 2022). The TMQ includes 20 items rated on a five-point Likert scale (0–4), yielding a total score range of 0–80. Scores were categorized as indicating no (0–35), low (36–45), moderate (46–53), or high (54–80) time management skills. The TMQ demonstrated acceptable internal consistency in this study (Cronbach’s *α* = 0.88) (Aeon, Faber & Panaccio, 2021).

#### Academic performance

The fourth section of the questionnaire included the validated Arabic version of the Academic Performance Scale (APS). This questionnaire consists of eight statements, each rated on a five-point Likert scale; “Strongly Agree” scored 5, “Agree” scored 4, “Neutral” scored 3, “Disagree” scored 2, and “Strongly Disagree” scored 1. The total score ranges from 0 to 40 and is categorized as follows: Excellent (33–40), Good Performance (25–32), Moderate Performance (17–24), Poor Performance (9–16) and Failing Performance (0–8). (Alsalem, Alamodi & Hazazi, 2017). This questionnaire was validated in different settings (Alhowimel et al., 2024; Zuljevic et al., 2022).

### Inclusion and exclusion criteria

All students enrolled in the selected classes during the 2023–2024 academic year were eligible to participate. Students with a prior diagnosis of ADHD, those receiving treatment for ADHD, individuals reporting substance use, and those currently using psychotropic medications were excluded.

### Handling of missing data

Questionnaires with missing responses exceeding 10% were excluded from the analysis. For questionnaires with minimal missing data (<10%), complete-case analysis was applied. No imputation methods were used.

### Statistical analysis

The collected data were analyzed using the Statistical Product and Service Solutions (SPSS) software, version 27. Descriptive statistics were employed to summarize the data, including means and standard deviations for continuous variables and frequencies and percentages for categorical variables. Differences between categories of independent variables were assessed using the Chi-square test. Logistic regression analysis was performed to identify factors independently associated with adult ADHD symptoms. Statistical significance was set at a two-tailed *P* value of < 0.05. All results were reported with corresponding 95% confidence intervals (CIs) to reflect both statistical significance and precision.

### Ethical consideration

The study protocol was reviewed and approved by the Standing Committee for Scientific Research at Jazan University (Reference No. REC-45/05/855). Participation in the study was voluntary, and informed consent was obtained from all participants. Participants’ confidentiality and anonymity were maintained throughout the study. The study was conducted in accordance with the ethical principles of the Declaration of Helsinki.

## Results

Table 1 shows the demographic data of participants who completed their questionnaires. Of the 763 students who received the questionnaire, 612 completed questionnaires were included in the final analysis, yielding a complete-response rate of 80.2%. Most participants were males (64.2%), and 80.6% reported sleeping 4–8 h per night. More than half of the participants (65.2%) had a GPA of 4.0 or higher the year before the study.

**Table 1:** Demographic characteristics, sleep duration and grade point average among students at Jazan University, Jazan, Saudi Arabia.

**Characteristic**	**Category**	**Frequency** **(*N* = 612)**	**Percentage**
Gender	Female	219	35.8%
	Male	393	64.2%
Age, mean ± SD (years)	–	21.04 ± 1.6	–
Weight, mean ± SD (kg)	–	65.51 ± 19.2	–
Height, mean ± SD (cm)	–	165.41 ± 9.4	–
College	Medicine	141	23.0%
	Nursing	158	25.8%
	Engineering	133	21.7%
	Business Administration	180	29.4%
Sleeping Hours	<4 h	46	7.5%
	4-8 Hours	493	80.6%
	>8 h	73	11.9%
Family Monthly Income	<5,000 SR	73	11.9%
	5,000–10,000	148	24.2%
	10,000–20,000	226	37.0%
	>20,000	164	26.8%
Mother’s Education	Illiterate	57	9.3%
	Primary education	87	14.2%
	Intermediate education	10	1.6%
	Secondary education	154	25.2%
	University-bachelor	200	32.7%
	Diploma	78	12.7%
	Postgraduate study	26	4.2%
Father’s Education	Illiterate	25	4.1%
	Primary education	50	8.2%
	Intermediate education	6	1.0%
	Secondary education	117	19.1%
	University-bachelor	288	47.1%
	Diploma	74	12.1%
	Postgraduate study	52	8.5%
Student’s GPA	<3	67	10.9%
	3.00–3.49	46	7.5%
	3.50–3.99	100	16.3%
	4.00–4.50	184	30.1%
	>4.5	215	35.1%

**Notes.**

Mean ± SD Mean value ± standard deviation GPA Grade Point Average

Figure 2 shows that 26% (159/612) of participants screened positive for adult ADHD symptoms. Figure 2 also shows that 59.5% of participants had low-to-high levels of time management skills. According to the APS, good academic performance was the most common category, reported by 49.3% of participants .

**Figure 2:**
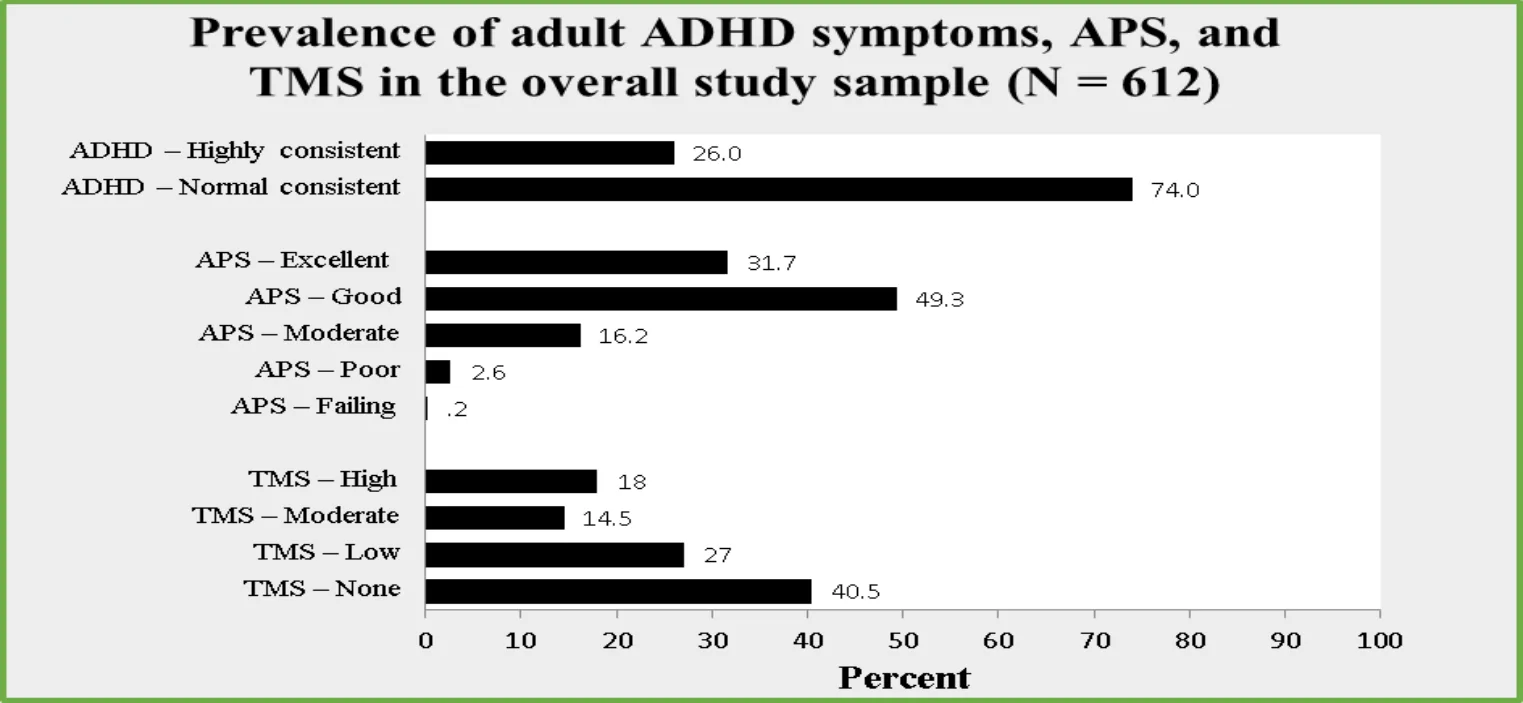
Prevalence of adult ADHD symptoms, APS, TMS (TMS) in the overall study sample (*N* = 612).

Figure 3 illustrates the distribution of academic performance status and time management skills among participants with ADHD symptoms. Good APS was the most prevalent category (56.0%), followed by moderate performance (21.4%) and excellent performance (19.5%). Poor performance was reported by 3.1% of participants, whereas no participants were classified as failing. Regarding time management skills, 42.1% of participants reported no time management skils (TMS), 26.4% reported low levels, 13.8% reported moderate levels, and 17.6% reported high levels.

**Figure 3:**
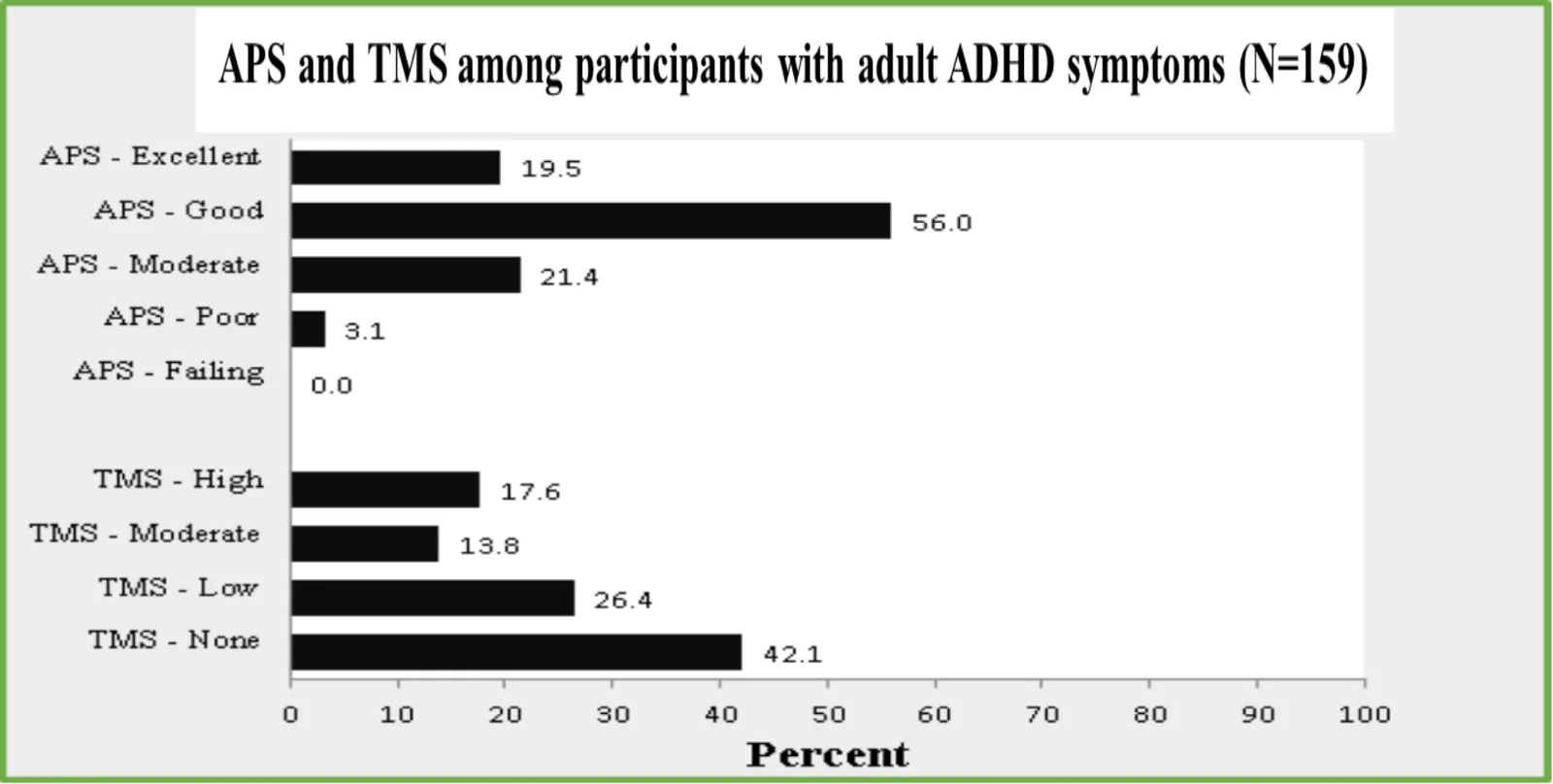
Distribution of the APS and TMS levels among participants with adult ADHD symptoms.

Table 2 shows the unadjusted bivariate relationship between different factors and the likelihood of exhibiting adult ADHD symptoms among Jazan University students. The factors significantly associated with ADHD symptoms were sleeping fewer than four hours per night, a GPA below 3.0, and poor or moderate academic performance. On the other hand, there were no significant differences in age or body mass index (BMI) between participants with adult ADHD symptoms and the nonclinical group (both *p* > 0.05).

**Table 2:** Demographic characteristics, sleep duration, time management strategy, and academic performance among Jazan University students in relation to adult ADHD symptoms status.

**Characteristic**	**Category**		**Adult ADHD** ** symptoms**				***P*-value**
		All (*N* = 612)	**No** **(*N* = 453)**		**Yes** **(*N* = 159)**		
			*n*	*Pct.*	*n*	*Pct.*	
Age (Years)	Mean ± SD	21.0 ± 1.6	21.0 ± 1.6		21.1 ± 1.7		0.817^^@^^
BMI (kg/m^2^)	Mean ± SD	23.7 ± 0.1	23.7 ± 0.1		23.9 ± 0.1		0.639^^@^^
Gender	Female	219	152	69.4%	67	30.6%	0.520
	Male	393	301	76.6%	92	23.4%	
College	Medicine	141	101	71.6%	40	28.4%	0.851^a^
	Nursing	158	116	73.4%	42	26.6%	
	Engineering	133	101	75.9%	32	24.1%	
	Business Administration	180	135	75.0%	45	25.0%	
Sleeping Hours	<4 h	46	26	56.5%	20	43.5%	0.019^a^^,^^*^
	4–8 Hours	493	372	75.5%	121	24.5%	
	>8 h	73	55	75.3%	18	24.7%	
Monthly Family Income	<5000 SR	73	56	76.7%	17	23.3%	0.710^a^
	5,000–1,000	148	105	70.9%	43	29.1%	
	10,000–20,000	226	171	75.7%	55	24.3%	
	>20,000	164	120	73.2%	44	26.8%	
Father’s Education	Illiterate	25	16	64.0%	9	36.0%	0.316^a^
	Primary education	50	37	74.0%	13	26.0%	
	Intermediate education	6	4	66.7%	2	33.3%	
	Secondary education	117	79	67.5%	38	32.5%	
	University (Bachelor)	288	217	75.3%	71	24.7%	
	Diploma	74	61	82.4%	13	17.6%	
	Postgraduate study	52	39	75.0%	13	25.0%	
Mother’s Education	Illiterate	57	38	66.7%	19	33.3%	0.174^a^
	Primary education	87	60	69.0%	27	31.0%	
	Intermediate education	10	9	90.0%	1	10.0%	
	Secondary education	154	123	79.9%	31	20.1%	
	University(Bachelor)	200	142	71.0%	58	29.0%	
	Diploma	78	62	79.5%	16	20.5%	
	Postgraduate study	26	19	73.1%	7	26.9%	
University GPA	<3	67	39	58.2%	28	41.8%	0.009^a^^,^^*^
	3.00–3.49	46	37	80.4%	9	19.6%	
	3.50–3.99	100	69	69.0%	31	31.0%	
	4.00–4.50	184	145	78.8%	39	21.2%	
	>4.5	215	163	75.8%	52	24.2%	
TMS category	No TMS	248	181	73.0%	67	27.0%	0.969^a^
	Low TMS	165	123	74.5%	42	25.5%	
	Moderate TMS	89	67	75.3%	22	24.7%	
	High TMS	110	82	74.5%	28	25.5%	
APS category	Failing Performance	1	1	100%	0	.0%	0.001^+^^,^^*^
	Poor Performance	16	11	68.8%	5	31.3%	
	Moderate Performance	99	65	65.7%	34	34.3%	
	Good Performance	302	213	70.5%	89	29.5%	
	Excellent Performance	194	163	84.0%	31	16.0%	

**Notes.**

@ Student *t*-test was used.

a Chi-Square Test was used.

+ Fisher Exact Test was used.

* Significant results at the 5% significance level.

Pct. percentages of the rows BMI Body Mass Index GPA Grade Point Average TMS Time Management Skills APS Academic Performance Scale

Table 3 shows the adjusted logistic regression analysis with coding for the categorical variables, with the reference group for each variable indicated by the corresponding row. For gender, the reference group is male. Females are 1.58 times more likely to have adult ADHD symptoms compared to males (95% CI [1.06–2.34]).

**Table 3:** Logistic regression analysis of factors associated with adult ADHD symptoms among Jazan University Students.

	B	*P*-value	Odds ratio of having adult ADHD symptoms	95% CI for OR	
				Lower	Upper
Female, ** ref: Male**	0.46	.022	1.58	1.07	2.34
Sleeping’s hours, ** ref.: >8 hours**		.027			
<4 h	0.90	.030	2.46	1.08	5.60
4–8 h	0.00	.995	1.00	0.56	1.80
University GPA, ** ref.: >4.5**		.016			
<3	0.72	.019	2.05	1.12	3.74
3.00–3.49	−0.28	.493	0.75	0.33	1.70
3.5–3.99	0.35	.215	1.41	0.82	2.44
4.00–4.50	−0.27	.269	0.76	0.47	1.24
APS, ** ref.: Excellent performance**		.003			
Failing + Poor Performance	0.69	.239	1.98	0.63	0.62
Moderate Performance	0.96	.001	2.62	1.46	4.71
Good Performance	0.82	.001	2.28	1.43	3.65

**Notes.**

APS was recoded into broader categories for regression analysis to avoid sparse-cell estimation problems. *P* values < 0.05 considered statistically significant.

For sleeping hours, the reference category is “>8 h.” The results indicate that individuals who sleep less than 4 h are 2.46 times more likely to have adult ADHD symptoms (95% CI [1.08–5.60]) compared to those who sleep more than 8 h.

Regarding GPA, the reference group is those with a GPA greater than 4.5. Individuals with a GPA of less than 3 are 2.1 times more likely to have adult ADHD symptoms (95% CI [1.12–3.74]) compared to those with a GPA higher than 4.5. In other words, lower GPA scores were associated with greater likelihood of adult ADHD symptoms.

The results in Table 3 also reveal that the APS is associated with the adult ADHD symptoms. As shown in the table, participants with “moderate performance” is 2.62 times more likely to have adult ADHD symptoms (95% CI [1.46–4.71]) compared to those with “excellent APS.” Similarly, participants with “good APS” have a 2.28 times higher likelihood of having adult ADHD symptoms (95% CI [1.43–3.65]) compared to those with “excellent APS.”

## Discussion

Our study at Jazan University identified a high prevalence of adult ADHD symptoms, with 26% of participating students exhibiting symptom levels consistent with the disorder. This estimate substantially exceeds global prevalence rates for adult ADHD, which typically range from 2.5% to 6.7% (Posner, Polanczyk & Sonuga-Barke, 2020; Song et al., 2021), as well as reported rates from Arab countries such as Lebanon (1.8%) and Iraq (0.6%) (Fayyad et al., 2017). These findings suggest a considerable burden of ADHD symptoms within the university population and underscore the need for increased awareness, early screening, and targeted support services in higher education settings.

Previous studies conducted in Saudi Arabia have reported wide variability in prevalence of adult ADHD symptoms estimates. For example, rates of 11.9% among university students and up to 46.6% in online adult samples have been documented (Alghamdi et al., 2022; Alharbi et al., 2023). Such heterogeneity likely reflects differences in study design, sampling strategies, population characteristics, and assessment tools. In the present study, a multistage random sampling approach was used to enhance representativeness across academic disciplines. Collectively, these findings suggest that adult ADHD symptoms may be more prevalent in Saudi Arabia than global averages, highlighting the need for further large-scale, methodologically rigorous investigations.

Female students in our study demonstrated a higher prevalence of adult ADHD symptoms (30.6%) compared with male students (23.4%). Logistic regression analysis indicated that female students had 1.58 times higher odds of screening positive for adult ADHD symptoms. Although ADHD has traditionally been reported as more prevalent among males (Faheem et al., 2022), accumulating evidence suggests that gender differences diminish in adulthood (Gilbert et al., 2023; Quinn & Madhoo, 2014; Skogli et al., 2013; Stibbe et al., 2020). Recent Saudi studies reporting higher prevalence among females further support these findings (Alghamdi et al., 2022; Alharbi et al., 2023).

No significant differences in the prevalence adult ADHD symptoms were observed across academic disciplines, indicating that adult ADHD affects students irrespective of field of study. Similarly, no significant association was identified between ADHD symptoms and time management skills. While several studies have reported impaired time management skills among individuals with ADHD (De la Charie et al., 2021; Mette, 2023), other research suggests that some individuals develop compensatory strategies that mitigate functional impairments (Champ, Adamou & Tolchard, 2021; Oguchi et al., 2021; Ptacek et al., 2019; Sedgwick-Muller et al., 2022; Yadav et al., 2021). These divergent findings likely reflect the heterogeneous presentation of ADHD and variability in coping mechanisms. Given that the subgroup presented in Fig. 3 consisted of students who screened positive for ADHD symptoms rather than individuals with a confirmed clinical diagnosis, it is plausible that some of them had already developed adaptive strategies, partially offsetting difficulties in TMS. However, time management skills were assessed using a single self-report measure; therefore, these findings should be interpreted cautiously.

Nevertheless, university students with adult ADHD symptoms may remain vulnerable to cycles of academic stress, dissatisfaction, and ineffective self-regulation (Kwon, Kim & Kwak, 2018). These challenges highlight the importance of institutional readiness to support students with adult ADHD. Universities may address these needs through academic accommodations, such as examination adjustments, structured tutoring, flexible learning modalities, and tailored support services. Additionally, interventions incorporating cognitive-behavioral therapy, coaching, and mindfulness have demonstrated effectiveness in supporting university students with adult ADHD (Alvarez-Godos, Ferreira & Vieira, 2023).

BMI was calculated to assess its association with adult ADHD symptoms (Table 2). No significant relationship was observed between BMI and self-reported ADHD symptoms among Jazan University students. Previous studies have reported an association between obesity and adult ADHD symptoms, potentially mediated by genetic factors (Karhunen et al., 2021; Pagoto et al., 2009). However, this association may be confounded by comorbid conditions such as depression (Sahan, Zengin Eroglu & Sertcelik, 2021) or by differences in genetic profiles (Karhunen et al., 2021). Consequently, the relationship between BMI and ADHD remains complex and incompletely understood (Cortese, 2019).

Sleep duration emerged as an important factor associated with adult ADHD symptoms. Students reporting fewer than four hours of sleep per night were significantly more likely to exhibit adult ADHD symptoms, underscoring the critical role of sleep in attentional regulation and cognitive functioning (French et al., 2023). Sleep deprivation may exacerbate adult ADHD symptoms and impair concentration, organization, and task completion, thereby negatively affecting academic functioning (Beebe, 2011; Cassoff, Wiebe & Gruber, 2012; Niermann & Scheres, 2014). These findings emphasize the need to promote healthy sleep practices among university students.

Academic performance was assessed using two validated measures, GPA and the APS, to enhance measurement validity. GPA provides an objective indicator of academic achievement; however, it may be influenced by variability in grading practices and assessment standards. In contrast, the APS captures both objective and subjective aspects of performance by incorporating self-reported engagement and perceived academic functioning, thereby addressing dimensions not fully reflected in examination scores (Crede & Kuncel, 2008; Jiang, Horta & Yuen, 2024; Richardson, Abraham & Bond, 2012). The combined use of GPA and APS therefore offers a more comprehensive assessment of academic performance.

Lower academic performance was significantly associated with adult ADHD symptoms. Students with a GPA below 3 were 2.09 times more likely to screen positive for adult ADHD symptoms than those with higher GPAs. These findings are consistent with previous research linking adult ADHD to poorer academic outcomes (Alghamdi et al., 2022; Birchwood & Daley, 2012; Gormley et al., 2019; Taylor & Zaghi, 2022), although some studies have reported null associations (Alsafar, Alsaad & Albukhaytan, 2024). While a recent systematic review reported a negative association between adult ADHD and academic achievement (Pagespetit et al., 2025), other studies found mixed or inconclusive results (Sönmez et al., 2023), likely due to methodological variability.

Using the APS, students with poor to moderate academic performance demonstrated higher adult ADHD symptom prevalence than those with excellent performance. However, a notable proportion of students with adult ADHD symptoms (29.5%) reported good academic performance. Logistic regression analysis revealed a complex pattern across performance categories, reflecting inconsistencies in the literature. These discrepancies may be attributable to differences in study design, assessment instruments, and sample characteristics (Arnold et al., 2020; Nordby et al., 2023). Furthermore, the university-based nature of the sample may partly explain these findings, as students who progress to higher education may have developed coping strategies that attenuate the academic impact of adult ADHD symptoms (Sedgwick-Muller et al., 2022).

Beyond core symptoms such as inattention and impulsivity, university students commonly experience stressors related to academic workload, extracurricular commitments, and personal responsibilities. These stressors may result in behaviors resembling adult ADHD symptoms, such as forgetfulness and disorganization, complicating accurate identification (AlZaben et al., 2018; Figueiredo et al., 2020; Zablotsky et al., 2019). Limited awareness of adult ADHD among healthcare professionals may further contribute to underdiagnosis and insufficient recognition of its academic impact (Kosheleff et al., 2023).

## Study Limitations

This study’s cross-sectional design allows for the identification of associations but precludes causal inference. The use of self-administered questionnaires may have introduced reporting bias. However, investigator assistance and expert review of the questionnaire were used to improve clarity and comprehension. Although multistage sampling improved representativeness, selection bias cannot be entirely excluded. Additionally, time management skills were assessed using a single instrument; the inclusion of multiple measures could improve sensitivity in future research.

## Strengths of the study

Strengths include the use of validated instruments, a relatively large and diverse university sample, multistage random sampling, and the simultaneous examination of adult ADHD symptoms, sleep duration, time management skills, and academic performance.

## Conclusion

This study demonstrates a substantially higher prevalence of adult ADHD symptoms among Saudi university students compared with global estimates. Female gender, insufficient sleep, and lower GPA were significantly associated with adult ADHD symptoms, whereas relationships with time management skills and APS-assessed academic performance were less consistent. These findings highlight the multifactorial nature of adult ADHD symptoms and underscore the need for targeted institutional interventions, including academic accommodations, sleep hygiene promotion, and mental health support services. Longitudinal and diagnostic-based studies are needed to further clarify these relationships and inform effective intervention strategies.

##  Supplemental Information

10.7717/peerj.21688/supp-1 Supplemental Information 1 Data

10.7717/peerj.21688/supp-2 Supplemental Information 2 Arabic Quesionnaire

10.7717/peerj.21688/supp-3 Supplemental Information 3 English Questionnaire

## Additional Information and Declarations

### Data Availability

The following information was supplied regarding data availability: The data is available in the Supplemental Files.
