# Supplementary material for: Prevalence of adult attention-deficit/ hyperactivity disorder symptoms among university students and their relation to sleeping duration, time management skills, and academic performance
Source: PeerJ. 2026 Oct 5;14:e21688. doi: 10.7717/peerj.21688 (PMC13648212; doi:10.7717/peerj.21688)
Supplement: Supplementary file 1 [file peerj-14-21688-s002.docx]

**وبائيات اضطراب فرط الحركة ونقص الانتباه لدى البالغين وعلاقتها بإدارة الوقت والأداء الأكاديمي بين طلاب جامعة جازان: دراسة مقطعية .خلال العام الدراسي 2023-2024**

الاستبيان

1- العمر (يكتب العمر بالسنوات وتهمل الكسور):..................

ضع علامة صح أمام إجاباتك في الأسئلة التالية:

2- الكلية:

[	] الطب

[	] كلية التمريض

[	] إدارة أعمال

[	] الهندسة

3- الجنس :

[	] ذكر

[	] أنثى

4- الوزن (بالكيلوجرام):.............................

5- الطول (بالسنتيميتر):............................

6- كم ساعة تنام في اليوم:

[	] أقل من 4 ساعات

[	] 4-8 ساعات

[	] أكثر من 8 ساعات

7- دخل الأسرة الشهري (بالريال السعودي):

[	] <5000

[	] 5000-10000

[	] 10000-20000

[	] >20000

8- المستوى التعليمي للأب:

[	] لا يقرأ ولا يكتب

[	] تعليم ابتدائي

[	] تعليم ثانوي

[	] الدبلوم

[	] جامعي-بكالوريوس

[	] دراسات عليا

[	] أخرى:....................

9- المستوى التعليمي للأم:

[	] لا يقرأ ولا يكتب

[	] تعليم ابتدائي

[	] تعليم ثانوي

[	] الدبلوم

[	] جامعي-بكالوريوس

[	] دراسات عليا

[	] أخرى:....................

10- المعدل الجامعي:

[	] < 3.00

[	] 3.00-3.49

[	] 3.50-3.99

[	] 4.00-4.50

[	] >4.50

| الأعراض  رجاء أجب على الأسئلة في الأسفل، قم بتقدير نفسك في كل معيار يتم عرضه باستخدام المقياس على الجانب الأيسر من الصفحة. عند إجابة كل سؤال ضع علامة صح الذي يوضح بشكل أفضل كيف كنت تشعر وتتصرف خلال الستة شهور السابقة. | | أبدا | نادراً | أحيانا | غالبا | كثيرا جدا |
| --- | --- | --- | --- | --- | --- | --- |
| 11 | إلى أي مدى تواجهك مشكلة الانتهاء من التفاصيل النهائية للمشروع بمجرد قيامك بالأجزاء الصعبة منه؟ |  |  |  |  |  |
| 12 | إلى أي مدى تجد صعوبة في ترتيب الأشياء عندما يكون عليك القيام بمهمة تحتاج إلى التنظيم. |  |  |  |  |  |
| 13 | إلى أي مدى يكون لديك مشاكل في تذكر المواعيد والالتزامات. |  |  |  |  |  |
| 14 | عندما يكون لديك مهمة تحتاج الكثير من التفكير، إلى أي مدى تتجنب أو تتأخر عن بدء العمل. |  |  |  |  |  |
| 15 | إلى أي مدى تململ أو تلوي يديك أو قدميك عندما يكون عليك أن تجلس لفتره طويلة. |  |  |  |  |  |
| 16 | إلى أي مدى تشعر بأنك نشط بشكل مفرط وأنك مدفوع لفعل الأشياء وكأنك تقاد بواسطة موتور. |  |  |  |  |  |
| 17 | إلى أي مدى ترتكب فيها أخطاء بسبب الإهمال عندما يكون عليك العمل في مشروع ممل أو صعب. |  |  |  |  |  |
| 18 | إلى أي مدى تجد صعوبة في الحفاظ على انتباهك عند القيام بعمل ممل أو متكرر. |  |  |  |  |  |
| 19 | إلى أي مدى تجد صعوبة في التركيز فيما يقوله إليك الآخرين حتى عندما يتحدثون إليك مباشرة. |  |  |  |  |  |
| 20 | إلى أي مدى تضع الأشياء في غير مكانها أو تجد صعوبة في العثور على الأشياء في البيت والعمل. |  |  |  |  |  |
| 21 | إلى أي مدى تصاب فيها بالتشتت بسبب الإزعاج أو النشاطات حولك؟ |  |  |  |  |  |
| 22 | إلى أي مدى تترك فيها مكانك ومقعدك في المقابلات والمواقف الأخرى عندما يكون متوقعا أن تظل جالسا. |  |  |  |  |  |
| 23 | إلى أي مدى تشعر فيها أنك متوتر أو متململ؟ |  |  |  |  |  |
| 24 | إلى أي مدى تجد صعوبة في أن تسترخي أو أن تحصل على الراحة عندما يكون لديك وقت لنفسك. |  |  |  |  |  |
| 25 | إلى أي مدى تجد نفسك تتحدث بشكل مفرط عندما تكون في مناسبات اجتماعية؟ |  |  |  |  |  |
| 26 | عندما تكون في محادثة، إلى أي مدى تجد نفسك تنهي جمل الأشخاص المتحدثين إليك قبل أن يتمكنوا من إنهائها بأنفسهم. |  |  |  |  |  |
| 27 | إلى أي مدى تجد صعوبة في انتظار دورك في المواقف عندما يكون مطلوبا أن تأخذ دورك. |  |  |  |  |  |
| 28 | إلى أي مدى تقاطع فيها الآخرين عندما يكونوا مشغولين؟ |  |  |  |  |  |
| 29 | أقرأ بشكل انتقائي، وأتصفح المواد حتى أجد ما هو مهم، ثم أحدده. |  |  |  |  |  |
| 30 | أقوم بإعداد قائمة بالمهام التي يجب إنجازها كل يوم. |  |  |  |  |  |
| 31 | أحتفظ بكل شيء في مكانه المناسب. |  |  |  |  |  |
| 32 | أقوم بترتيب المهام التي يجب على القيام بها حسب أهميتها واستعجالها. |  |  |  |  |  |
| 33 | أنا أقوم بمهام بسيطة متعددة في نفس الوقت لكن أصب معظم تركيزي على المهمة الأساسية. |  |  |  |  |  |
| 34 | أقوم بإعداد قائمة بالمهام القصيرة التي تستغرق خمس دقائق أو عشر دقائق للقيام بها. |  |  |  |  |  |
| 35 | أقسم المشاريع الكبيرة إلى مراحل أصغر ومنفصلة. |  |  |  |  |  |
| 36 | أراجع جدول مهامي كل يوم بعد إنجاز مهامي. |  |  |  |  |  |
| 37 | أقوم بأهم المهام في أفضل وقت لي خلال اليوم. |  |  |  |  |  |
| 38 | لدي بعض الوقت خلال كل يوم يمكنني فيه العمل دون انقطاع. |  |  |  |  |  |
| 39 | أفعل اليوم ما يجب فعله. لا أؤجل الأشياء. |  |  |  |  |  |
| 40 | أقوم بتقييم استخدام وقتي بشكل دوري أثناء استخدام جدولي الدراسي. |  |  |  |  |  |
| 41 | أضع مواعيد نهائية لنفسي إذا لم تُحدد لي. |  |  |  |  |  |
| 42 | أفعل شيئًا مفيدًا كلما كنت في انتظار شيء ما. |  |  |  |  |  |
| 43 | أتجنب هدر الوقت. |  |  |  |  |  |
| 44 | أنهي شيئًا واحدًا على الأقل كل يوم. |  |  |  |  |  |
| 45 | أخصص بعض الوقت خلال اليوم للوقت الشخصي (للتخطيط، التأمل، التمارين). |  |  |  |  |  |
| 46 | أضع لنفسي أهدافًا للسنة الأكاديمية. (أكاديمية، شخصية، روحية) - طويلة المدى. |  |  |  |  |  |
| 47 | أضع لنفسي أهدافًا كل فصل دراسي. (أكاديمية، شخصية، روحية ... إلخ). |  |  |  |  |  |
| 48 | أحاول باستمرار إيجاد طرق بسيطة لاستخدام وقتي بشكل أكثر كفاءة. |  |  |  |  |  |

يرجى الإجابة على كل سؤال من الأسئلة القادمة باختيار إجابة واحدة من المقياس المكون من 5 نقاط بحيث يعكس بدقة ما تفعله أو فعلته كطالب. كن صادقًا قدر الإمكان لأنه يمكن استخدام المعلومات لاكتشاف مجالات القوة.

| الاسئلة | موافق بشدة | موافق | محايد | غير موافق | غير موافق بشدة |
| --- | --- | --- | --- | --- | --- |
| 49- أجهز نفسي جيدا في كل موادي. |  |  |  |  |  |
| 50- أستمع وأركز جيدا في كل مناقشة. |  |  |  |  |  |
| 51- أريد الحصول على درجات جيدة في كل مادة. |  |  |  |  |  |
| 52- أشارك بفاعلية في كل مادة. |  |  |  |  |  |
| 53- أبدأ بالأوراق والواجبات والمشاريع فورا عند إقرارها. |  |  |  |  |  |
| 54- أستمتع بالواجبات المنزلية والأنشطة في كل مادة لأنها تساعدني على تطوير مهاراتي. |  |  |  |  |  |
| 55- أبذل المزيد من الجهد عندما أقوم بالمهام والواجبات الصعبة. |  |  |  |  |  |
| 56- حل المشكلات هواية مفيدة بالنسبة لي. |  |  |  |  |  |

57- هل خضعت لعلاج لمرض قصور الانتباه وفرط الحركة (ADHD) من قبل؟:

(نعم) [	]

(لا) [	]
