# Supplementary material for: Prevalence of adult attention-deficit/ hyperactivity disorder symptoms among university students and their relation to sleeping duration, time management skills, and academic performance
Source: PeerJ. 2026 Oct 5;14:e21688. doi: 10.7717/peerj.21688 (PMC13648212; doi:10.7717/peerj.21688)
Supplement: Supplementary file 2 [file peerj-14-21688-s003.docx]

**Adult ADHD in relation to time management and academic performance among university students: a cross-sectional study**

Questionnaire:

1- Age (Write age in years and ignore fractions):..................

Mark your answers to the following questions:

2- College:

[ ] Medicine

[ ] College of Nursing

[ ] Business Administration

[ ] Engineering

3- Gender:

[ ] Male

[ ] Female

4- Weight (in kilograms):.............................

5- Height (in centimeters):.............................

6- How many hours do you sleep per day:

[ ] Less than 4 hours

[ ] 4-8 hours

[ ] More than 8 hours

7- Monthly family income (in Saudi Riyals):

[ ] <5,000

[ ] 5,000-10,000

[ ] 10,000-20,000

[ ] >20,000

8- Father's educational level:

[ ] Illiterate

[ ] Primary Education

[ ] Secondary Education

[ ] Diploma

[ ] University-Bachelor's

[ ] Postgraduate Studies

[ ] Other: ....................

9- Mother's Educational Level:

[ ] Illiterate

[ ] Primary Education

[ ] Secondary Education

[ ] Diploma

[ ] University-Bachelor's

[ ] Postgraduate Studies

[ ] Other: ....................

10- University GPA:

[ ] < 3.00

[ ] 3.00-3.49

[ ] 3.50-3.99

[ ] 4.00-4.50

[ ] >4.50

| Very Often | Often | Sometimes | Rarely | Never | Symptoms  Please answer the questions below, rating yourself on each of the criteria shown using the scale on the right side of the page. As you answer each question, place an X in the box that best describes how you have felt and conducted yourself over the past 6 months | |
| --- | --- | --- | --- | --- | --- | --- |
|  |  |  |  |  | How often do you have trouble wrapping up the final details of a project, once the challenging parts have been done? | 11 |
|  |  |  |  |  | How often do you have difficulty getting things in order when you have to do  a task that requires organization? | 12 |
|  |  |  |  |  | How often do you have problems remembering appointments or  obligations? | 13 |
|  |  |  |  |  | When you have a task that requires a lot of thought, how often do you avoid or delay getting started? | 14 |
|  |  |  |  |  | How often do you fidget or squirm with your hands or feet when you have to sit down for a long time? | 15 |
|  |  |  |  |  | How often do you feel overly active and compelled to do things, like you were driven by a motor? | 16 |
|  |  |  |  |  | How often do you make careless mistakes when you have to work on a boring or difficult project? | 17 |
|  |  |  |  |  | How often do you have difficulty keeping your attention when you are doing boring or repetitive work? | 18 |
|  |  |  |  |  | How often do you have difficulty concentrating on what people say to you, even when they are speaking to you directly? | 19 |
|  |  |  |  |  | How often do you misplace or have difficulty finding things at home or at work? | 20 |
|  |  |  |  |  | How often are you distracted by activity or noise around you? | 21 |
|  |  |  |  |  | How often do you leave your seat in meetings or other situations in which you are expected to remain seated? | 22 |
|  |  |  |  |  | How often do you feel restless or fidgety? | 23 |
|  |  |  |  |  | How often do you have difficulty unwinding and relaxing when you have time to yourself? | 24 |
|  |  |  |  |  | How often do you find yourself talking too much when you are in social situations? | 25 |
|  |  |  |  |  | When you’re in a conversation, how often do you find yourself finishing the sentences of the people you are talking to, before they can finish them themselves? | 26 |
|  |  |  |  |  | How often do you have difficulty waiting your turn in situations when turn taking is required? | 27 |
|  |  |  |  |  | How often do you interrupt others when they are busy? | 28 |
|  |  |  |  |  | I read selectively, skimming the material until I find what is important, then highlighting it. | 29 |
|  |  |  |  |  | I make a list of tasks to accomplish each day | 30 |
|  |  |  |  |  | I keep everything in its proper place. | 31 |
|  |  |  |  |  | I prioritize the tasks I have to do according to their importance and urgency. | 32 |
|  |  |  |  |  | I concentrate on only one important task at a time, but I do multiple trivial tasks at once. | 33 |
|  |  |  |  |  | I make a list of short five- or ten-minute tasks to do. | 34 |
|  |  |  |  |  | I divide large projects into smaller, separate stages. | 35 |
|  |  |  |  |  | I review my planner each day after I complete my tasks. | 36 |
|  |  |  |  |  | I do the most important tasks at my best time during the day. | 37 |
|  |  |  |  |  | I have some time during each day when I can work uninterrupted. | 38 |
|  |  |  |  |  | I do today what needs to be done. I don’t procrastinate. | 39 |
|  |  |  |  |  | I periodically evaluate the use of my time while using my academic calendar. | 40 |
|  |  |  |  |  | I set deadlines for myself if they are not provided for me | 41 |
|  |  |  |  |  | I do something productive whenever I am waiting. | 42 |
|  |  |  |  |  | I avoid time wasters. | 43 |
|  |  |  |  |  | I finish at least one thing every day. | 44 |
|  |  |  |  |  | I schedule some time during the day for personal time alone (for planning, meditation, prayer, exercise). | 45 |
|  |  |  |  |  | I set goals for the academic year. (Academic, Personal, Spiritual) –Long Term | 46 |
|  |  |  |  |  | I set goals for myself each semester. (Academic, Personal, Spiritual…etc.) | 47 |
|  |  |  |  |  | I continually try to find little ways to use my time more efficiently. | 48 |

Please answer each of the following questions by selecting one answer from a 5-point scale that accurately reflects what you do or have done as a student. Be as honest as possible, as this information can be used to identify areas of strength.

| Strongly disagree | Disagree | Neutral | Agree | Strongly agree | Questions |
| --- | --- | --- | --- | --- | --- |
|  |  |  |  |  | 49- I made myself ready in all my subjects. |
|  |  |  |  |  | 50- I pay attention and listen during every discussion. |
|  |  |  |  |  | 51- I want to get good grades in every subject. |
|  |  |  |  |  | 52- I actively participate in every discussion. |
|  |  |  |  |  | 53- I start papers and projects as soon as they are assigned. |
|  |  |  |  |  | 54- I enjoy homework and activities because they help me improve my skills in every subject. |
|  |  |  |  |  | 55- I exert more effort when I do difficult assignments. |
|  |  |  |  |  | 56- Solving problems is a useful hobby for me. |

57- Have you ever received treatment for attention deficit hyperactivity disorder (ADHD)?:

(Yes) [ ]

(No) [ ]
